# Analysis of Sport Supplement Consumption by Competitive Swimmers According to Sex and Competitive Level

**DOI:** 10.3390/nu14153218

**Published:** 2022-08-06

**Authors:** Berta Moreno, Santiago Veiga, Antonio J. Sánchez-Oliver, Raúl Domínguez, Esther Morencos

**Affiliations:** 1 Exercise Physiology Group, Exercise and Sport Sciences, Faculty of Health Sciences, Universidad Francisco de Vitoria, 28223 Madrid, Spain; 2 Faculty of Physical Activity and Sports Sciences (INEF), Universidad Politécnica de Madrid (UPM), 28040 Madrid, Spain; 3 Departamento de Motricidad Humana Rendimiento Deportivo, Universidad de Sevilla, 41013 Sevilla, Spain

**Keywords:** nutrition, swimming, ergogenic aids, performance, elite swimmers

## Abstract

Sports supplements (SS) are commonly used by athletes to improve their performance. SS use by competitive swimmers is reported to be prevalent but there is no evidence of such use by elite swimmers, either male or female. The objective of this research was to study the patterns of SS use by competitive swimmers based on sex and competitive levels (national and international); Methods: Using the categories of the Australian Institute of Sport (AIS), a total of 102 competitive swimmers (59 men and 43 women) completed a validated self-administered questionnaire on the use of SS; (3) Results: Overall, 86.9% of swimmers had consumed SSs with no differences observed between males and females (*p* = 0.247) or between competitive levels (*p* = 0.597). The SS that were most consumed by swimmers were caffeine (53.5%), sport drinks (52.5%), sport bars (51.5%), and vitamin C (43.4%). SSs categorized as medical supplements were consumed significantly more frequently by international swimmers (*p* = 0.012), with significant differences also found in the level—sex interaction (*p* = 0.049); (4) Conclusions: Compared to other sports disciplines, the prevalence of SS consumption is high in competitive swimmers regardless of performance level or gender. However, the consumption of medical supplements was greater in swimmers at a higher performance level.

## 1. Introduction

The main objective of competitive swimming is to complete a set distance (ranging from 50 m to 1500 m in official swimming pool events) in the shortest possible time using the front crawl, breaststroke, backstroke, or butterfly technique [1]. Swimming events can last from approximately 20 s to 16 min approximately, and depending on the characteristics of the event, the efforts require different energy systems, such as high-energy phosphagen, glycolytic and oxidative phosphorylation of carbohydrates, fats, or proteins [2]. 

The fact that swimming takes place in the water poses unique challenges for the swimmer, with the obligation to reduce drag forces and to maximize propulsive forces becoming a decisive factor in dictating the physiological and energetic demands rather than the duration of the test itself [3]. Training programs for elite competitors usually consist of a large amount of high-intensity training [4,5], although specific training demands depend on the characteristics of the sprint, middle-distance, or distance event. For example, the aerobic involvement in a 400 m front crawl event is 81%, while in the shorter events, such as 50 m and 100 m front crawl, the aerobic contribution is 15.3% [2]. This also has consequences on the different nutritional requirements for success in each discipline [2,6,7].

Previous studies have investigated strategies to increase performance by means of better recovery [2,3,8,9,10] through the use of nutritional measures, such as sports supplements [8,11,12]. The SSs should be considered as a supplement to the usual diet, which is consumed with the aim of achieving specific performance benefits [13]. Despite this, only a small portion of current SSs on the market have been shown to produce significant improvements in performance [13,14,15,16]. Some SS, such as sodium citrate or phosphate, have been reported to provide a negligible benefit to exercise capacity [17,18,19]. Thus, it is necessary to carry out a cost—benefit analysis based on the criteria for SS safety, efficacy, and legality [15] prior to introducing supplements, providing strategies that allow us to resist in the face of external agents [20].

Different international institutions periodically publish position stands that are elaborated to guide the practice of supplementation, such as the Australian Institute of Sport (AIS) which created the ABCD system, whereby each SS is classified according to the level of scientific evidence [21]: SSs in group A present a high level of scientific evidence; those in group B can have a positive effect in certain circumstances, but more evidence is necessary; those in group C are supplements for which evidence is lacking; and those in group D are prohibited substances. At the same time, SSs are subdivided into sports foods, medical supplements, and ergogenic aids. Several studies have reported the effects of supplementation in athletes based on this classification system [14,22,23,24,25], and athletes following a SS program under the AIS guide have displayed a greater consumption of SSs with a high level of scientific evidence [26].

The reported range of SS consumption in sports is very wide and comprises between 30–95% of athletes [23,25,27]. Sex and level of performance are among the most important variables for SS consumption, as SS consumption increases with the competitive level of athletes and in men compared to women [13,23,25,28]. A higher rate of SS consumption has been reported among swimmers (in comparison to other disciplines) as swimming was ranked among the top four sports in terms of the SS use prevalence at the 2000 Sydney Olympic Games [29,30]. The SS consumption patterns in competitive swimmers indicated that all 23 swimmers surveyed in Sri Lanka had consumed SSs and had a daily intake of 3.4 different supplements [26]. However, these data were collected from swimmers with a low competitive level, and, to the best of our knowledge, no other evidence on the SS consumption prevalence or SS characteristics in elite swimmers has been observed.

Therefore, the objective of this study was to analyze the use of SSs specifically in competitive swimmers (national and international levels) and to describe the consumption pattern according to sex and competitive level. It was hypothesized that swimmers of a higher level of performance and male swimmers would present higher SS consumption.

## 2. Materials and Methods

### 2.1. Participants

A total of 102 competitive swimmers (59 men and 43 women) voluntarily participated in this investigation. All participants belonged to a federated swimming club and were competing in national (*n* = 60) and/or international (*n* = 39) events at the time. Swimmers of international level had taken part in international-level competitions with the national team for at least two years. Three swimmers from the original sample were eliminated from the study because they did not have the required competitive national level; therefore, 99 swimmers were included in the final sample. All participants had an average of six days of training per week, four hours of training per day, and an average of three days per week of out-of-water training.

### 2.2. Instruments

The questionnaire used contained three main sections: the first one collected anthropometric, personal, and social data of the respondent; the second section covered the practice of the sport activity and included 10 questions about, for example, the number of years federated, the competitive level, years competing in the national team, the hours of water training by week, the sessions of water training by week or dry-land training sessions by week. The last and most extensive section was related to SS consumption. This section contained three questions on the type of diet the swimmers had, 12 questions on the use and consumption of SS (i.e., what supplements are taken, for what reasons are they taken, who advises to take them, when are they taken, what were the reasons or where can the supplements be purchased), and, finally, two questions on the use of banned substances. In addition, this part included the definition of sports supplements (SS) proposed by Knapik [28] and an updated list of SSs. The response options included a time frame such as whether swimmers took SSs for training, competition, or both and whether they took them before, during, or after training and/or competition. 

The questionnaire was previously validated in terms of its content, application, structure, and presentation [31] and its quality was evaluated in a systematic review [28]. According to an eight-point scale that included assessments of sampling methods, sampling frame, sample size, measurement tools, bias, response rate, statistical presentation, and description of the participant sample, the quality of the questionnaire reached a 54% score (57 out of 164 questionnaires evaluated) and it was considered adequate to obtain accurate information on supplement use by athletes. It is worth noting its use in different studies that have analyzed the consumption of SS in athletes [14,24,25,27,32]. 

### 2.3. Process

All swimmers who participated in this research did so voluntarily and only had to complete a questionnaire on the use of supplements [31]. Swimmers completed the questionnaire between April and October 2019 in a web format that was prepared to facilitate its distribution (Google Forms, Google, Mountain View, CA, USA). The information recorded in the questionnaires did not allow the identification of the swimmers, all of whom remained anonymous. The participants were recruited with the help of the National Swimming Federation, which distributed an e-mail with instructions for completion and an online version of the questionnaire to all the teams with a national license. Thus, when the swimmers went to their usual training pool, the characteristics of the study were explained to them, and consent was obtained from all of them. The protocol complies with the Declaration of Helsinki for human research and was approved by the Ethics Committee of the local university committee.

### 2.4. Statistical Analysis

The Kolmogorov–Smirnov test and the Levene test were applied to check for normality and homoscedasticity. Quantitative variables were presented as an average (M) ± standard deviation (SD), while qualitative variables were in percentages. For the analysis of possible differences in the level of performance (international vs. national) and of the possible differences in sex with regard to the motivation, expectations, and contextualization of the use of SS, a chi-square test (χ2) was performed. If statistical differences were reported, an odds ratio (OR) was also performed. As to the total SSs ingested, a Student’s T-test for independent samples was carried out to analyze possible differences between the levels of performance or between performance based on sex. The statistical level of significance was set at *p* < 0.05. The statistical analyses were performed using the Statistical Package for Social Sciences (version 18.0 for Mac, SPSSTM Inc., Chicago, IL, USA).

## 3. Results

The sample characteristics are reported in Table 1. Male swimmers presented as taller (*p* < 0.001) and heavier (*p* = 0.005) than female swimmers, although no anthropometric differences were reported for level or level—sex. Additionally, no statistically significant differences were found for the number of weekly training days or the number of strength training days in relation to the competitive level, sex, or level—sex (*p* > 0.05). However, national female swimmers performed more strength training days than did international female swimmers (*p* = 0.044). No differences were reported for level (*p* = 0.522) and sex (*p* = 0.384) in the years of federated swimming with 20.2% of the sample federate for less than 5 years, 39.4% between 5 and 8 years, and 40.4% for more than 9 years.

### 3.1. SS Consumption Characteristics

In relation to SS consumption characteristics, 6.1% of the sample said they were against SS consumption in swimming, while 80.8% said they were in favor and 13.1% either did not know or did not answer, with no statistically significant differences observed according to sex (*p* = 0.841) or competitive level (*p* = 0.417). When asked if they had ever consumed SSs, 86.9% of the sample answered affirmatively, with no differences observed between men and women (*p* = 0.247) or between swimmers at international and national levels (*p* = 0.597). The main motivations for SS consumption were performance improvement (59.2%), health care (14.6%), or to cover dietary deficits (10.0%), with no statistically significant differences between sexes (*p* = 0.109) or between the level of swimmers (*p* = 0.530).

**Figure 1:**
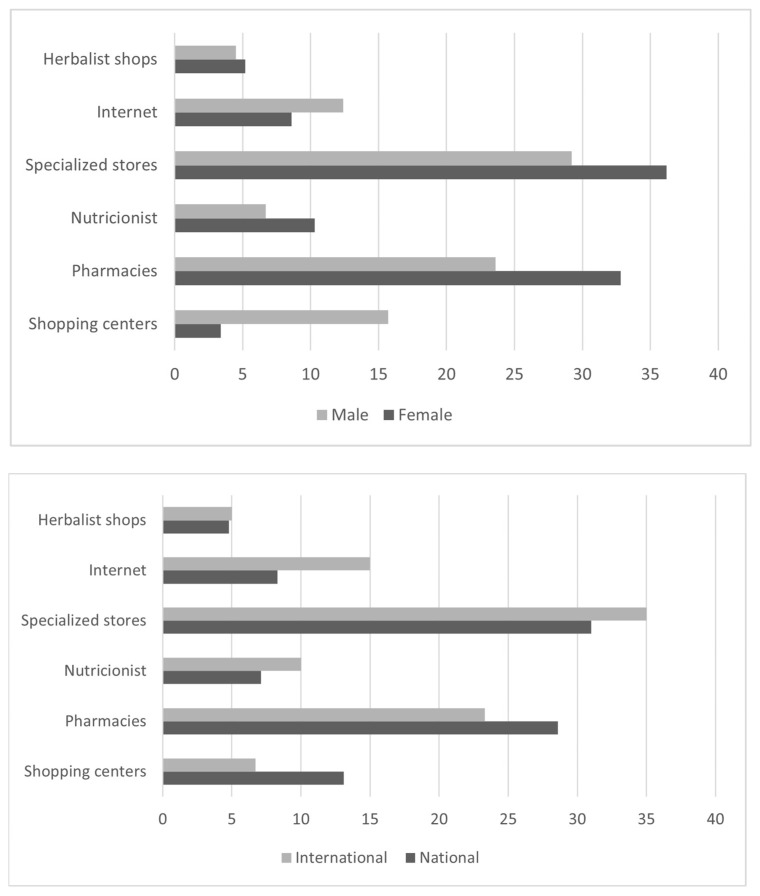
Main site of supplement purchase by elite swimmers according to sex (**up**) and competitive level (**down**).

As shown in Figure 1, SSs were most frequently purchased at specialized SS stores (32.0%) and pharmacies (27.2%), followed by shopping centers (10.9%) and the Internet (10.9%), with no differences between sexes (*p* = 0.193). There were also no differences between swimmers at the national and international levels (*p* = 0.822). 

**Figure 2:**
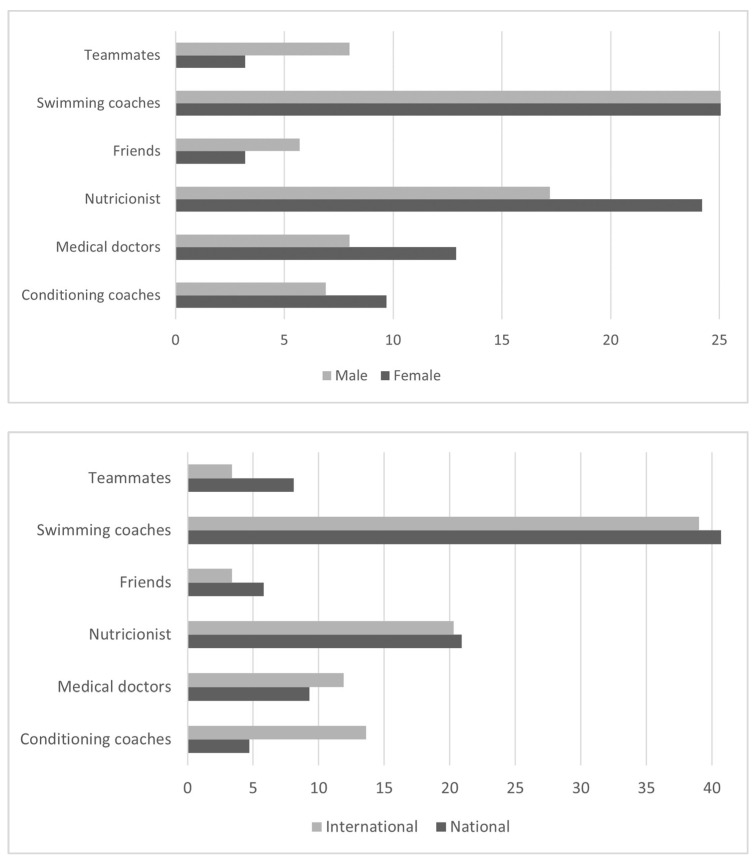
Sources of information or advice on the use of supplements by elite swimmers according to sex (**up**) and competitive level (**down**).

As can be seen in Figure 2, the coach was the person who most frequently advised the consumption of SSs (40.3%), followed by the dietitian—nutritionist (20.1%), the physician (10.0%), the physical trainer (8.1%), family (6.7%), teammates (6.0%), friends (4.7%), or others (4.1%). No significant differences were observed between sexes (*p* = 0.421) or levels (*p* = 0.227).

Regarding when SSs were consumed within the season, most subjects reported consuming SSs during the competitive period (66.0%), followed by those who consumed them only during the training period (23.0%), throughout the year (7.0%), or at other times (4.0%), with no differences found according to sex (*p* = 0.702) or competitive level (*p* = 0.838). In addition, no differences were found between men and women (*p* = 0.284), although differences were observed according to level (*p* < 0.001). International level swimmers presented a greater mode of SS consumption before, during, and after exercise (39.5% vs. 18.6%), whereas national level swimmers had a greater SS consumption mode exclusively before (42.4% vs. 13.2%; OR = 2.56 [1.05–6.27]) or after (27.2% vs. 21.1%; OR = 1.29 [0.60–2.74]) exercise as compared to international level swimmers.

### 3.2. Type of SS Consumption

Group-level evidence of SSs (A, B, and C; see Table 2) showed no statistical differences in SS consumption for competitive level, sex, or level—sex (*p* > 0.05). As for the different subgroups of group A, a higher consumption of medical supplements was found in international level swimmers (*p* = 0.012), reaching statistically significant differences in men (*p* = 0.001) but not in women (*p* = 0.709). In addition, higher consumption was found in women at the national level as compared to men at the same competitive level (*p* = 0.013). Finally, the subgroup “sport performance” showed higher consumption for national level men as compared to national level women (*p* = 0.036).

As can be seen in Table 3, there was a consumption rate higher than 10% for 17 SSs in total, with caffeine (53.5%), sports drinks (52.5%), sports bars (51.5%), and vitamin C (43.4%) as the most consumed substances. No differences were observed based on sex for supplements with a consumption rate of at least 10%. Males were observed to have significantly lower consumption of vitamin complexes compared to females (*p* = 0.029; OR = 0.33 [0.12–0.87]), but they had a higher consumption of creatine (*p* = 0.041; OR = 2.92 [1.10–7.72]), taurine (*p* > 0.001), and a trend toward significance in the case of whey protein (*p* = 0.059; OR = 2.77 [0.99–7.7]). On the other hand, international level athletes presented a greater iron intake (*p* < 0.001; OR = 5.77 [2.32–14.32]) than those at the national level.

## 4. Discussion

The aim of this study was to analyze SS use in international and national level swimmers, describing the pattern of consumption according to sex and competitive level. Although previous studies [8,26,33,34,35] have examined SS consumption in competitive swimmers, to our knowledge, this is the first study to report on SS consumption in an extensive sample of elite level swimmers. The results of the present study show a high prevalence of SS use in competitive swimmers (86.9%) compared to other disciplines (30–95%), such as elite sailors (52%) [14] or rugby players (65.3%) [25]. These data support a higher use of SS in individual versus team sports (81% versus 58%) [36] and a prevalence similar to that of previous research on swimming [8,28,30,33,35,36].

### 4.1. Characteristics of SS Consumption in Competitive Swimmers

High training volume of competitive swimmers including the large number of weekly training sessions in and out the water [4] could explain the high prevalence of SS use in competitive swimmers. Contrary to expectations, no significant differences were observed between national and international level swimmers in the rate of SS consumption as has been shown in other studies [14,22]. This could be explained by the similar training volume of national and international caliber athletes, as defined by Mckay et al. [37] who distinguished the elite-international and the highly trained-national by the competitive standard. Finally, no differences were observed in the prevalence of SS use according to sex, contrary to other recent studies [14,25,36] but in line with similar training volume in swimming between gender.

The main reason for SS consumption in the present sample was to improve sports performance (59.2%), regardless of gender or competitive level. This motive for SS consumption is common (45–77.8%) among elite athletes in different sports [14,32,36,38], and it is consistent with the most used type of SS in the sample, that is, caffeine, which has previously been reported to improve sports performance [21,39]. The most frequent places of purchase of SS in this study, which were well beyond the rest, were specialized shops (32%) and pharmacies (27.2%), a finding that is consistent with a recent study of elite sailors by Caraballo et al. [14]. It is worth noting that online shopping, a current trend reported in some studies [25,36], was not one of the most frequent places of purchase considering the cases of biased and unreliable information, inclusion of undeclared pharmacological substances on the label, and lack of specific legislation [27,40,41,42,43].

Although the source of information about SS should be professionals in the field (sports doctors and/or dieticians—nutritionists), swimmers in the present research chose coaches as the main source of information (40.3%) as in a very recent study in gym users by Finamore [44] and the findings in a study of German athletes [45]. This demonstrates some areas of improvement in SS consumption by competitive swimmers, since athletes who receive advice from a professional have a higher consumption of SS for which there is a high level of evidence [25]. It is noteworthy that most of the SS consumed were of category A, which suggests that the coaches of our swimmers were very well informed and instructed to advise on responsible consumption of SS to elite swimmers. 

Most subjects reported consuming SS during the competitive period (66.0%), regardless of gender or level of competition. This is related to the three most consumed SS substances of caffeine, sports drinks, and sports bars as they tend to be used during competitive periods. The timing of SS consumption coincided with data reported in similar studies of elite athletes [14], although differences were observed between international and national swimmers. Higher level competitive swimmers had higher SS intake during exercise compared with the higher pre-exercise SS intake of lower level swimmers. The social environment, training regimens, and competition place great demands on elite athletes [4]; perhaps for this reason, added to the demands of the competition itself, means that the highest-level swimmers decide to resort to supplementation as an aid at the moment of greatest sporting demand. This finding in the present investigation is interesting since none of the other studies on swimmers has measured the moment of SSs [2].

The amount of SS consumed in the sample of competitive swimmers (5.45) was higher than what has been reported on fencers (3.33) [23], rugby players (3.9) [25], and sailors (3.9) [14] but lower than studies conducted with squash players (8.4) [32], rowers (16.28) [22], all elite athletes. These data support the hypothesis that there is a difference in consumption depending on the sport or sport modality [13,28,31], although we did not detect a lot of differences in SS consumption by sex [25] or competitive level [28]. 

### 4.2. Type of SS Consumption in Competitive Swimmers

When dividing SSs according to the level of evidence (AIS classification), national level men in group A had higher consumption than did women. Although women consumed more SSs in medical supplement subgroup A (0.4 vs. 1.0), these differences in consumption could be explained by the biggest differences in the sport performance subgroup (1.5 vs. 0.8). On the other hand, within the medical supplement subgroup, higher consumption was observed at the international versus the national level for men and at the national level for women as compared to men. The reason for these differences could be the inclusion of iron in the medical supplement subgroup as it has been reported to be the most consumed supplement by elite athletes in different sports [22,23,28,32,36] and specifically by women [28,36]. Iron deficiency is one of the most prevalent nutritional deficiencies in the athlete population, especially in women due to the increased demand for iron during menstruation [46,47]; it is also prevalent at the end of the competitive period [48], which is more demanding for international athletes. In addition, this supplement requires individual dispensing and appropriate sports physician/scientist supervision, so it is reasonable that international swimmers would have a higher intake.

Regarding the most consumed SS in the sample, differences were found according to sex, with men consuming more creatine and taurine and showing a tendency in the case of whey protein, and women consuming more vitamin complexes. These findings are related to those obtained in previous studies in which iron and vitamin consumption were higher for women and protein supplements (including creatine) were higher for men [28,49,50]. It is worth noting that most of the SSs consumed in the present research belonged to group A (more than 10% prevalence), which may suggest that the sample of swimmers was well informed about the most effective supplements in their sport, even if coaches were the main advisers about SSs, as mentioned above. For this reason, a good coach’s training-education in this subject (with documents such as the IOC, AIS, or ISSN [13,21,51]) could be a key aspect to correctly advise their swimmers at these levels.

Finally, the present study may present certain limitations that need to be noted for a better interpretation of the results. First, the questionnaire which was used to assess the consumption of dietary supplements in elite athletes collected information retrospectively, and this could lead to inaccurate information on the number and/or type of supplements reported. Even so, although the questionnaire collects the information retrospectively, a validated and reliable questionnaire was used to assess the use of dietary supplements in athletes, as was conducted in other studies of other sports with such a questionnaire [31]. These studies are proof that, despite these small limitations, such a questionnaire should not affect your answers, guaranteeing their accuracy. Second, it is possible that swimmers could have employed supplements but did not recognize their use at that time and, therefore, did not report their use in the questionnaire, resulting in a false negative.

The analysis of SS consumption in this study was analyzed per unit, not per quantity. The quantity should be subject to the weight of the athlete, so it is a very interesting topic for future studies. 

## 5. Conclusions

The present study indicated a high prevalence of SS consumption in competitive swimmers compared to other disciplines, with caffeine, sports drinks, and sports bars being the most consumed SSs. Swimmers reported performance enhancement as the primary motivation for SS consumption and, consequently, SS intake was most common during the competitive period. Differences in SS consumption were observed according to the competitive level, with international level swimmers exhibiting (i) a greater consumption of SSs during exercise (and not only before or after exercise) and (ii) a greater intake of medical supplements (like iron) with a higher level of scientific evidence. In addition, differences were observed by gender in the consumption of certain SSs such as creatine and taurine or the vitamin complexes. These results provide the first evidence of SS consumption in elite swimmers and demonstrate some specific patterns according to the high demands of volume and intensity in the elite swimming training programs.

## Figures and Tables

**Table 1:** Characterization of sample data divided by sex and competitive level (international and national).

Measure	Sex	International	National	*p*-Value Level	*p*-Value Sex	*p*-Value Level-Sex
Height (m)	Females	1.69 ± 0.06 ^λ^	1.68 ± 0.07 ^λ^	0.380	<0.001	0.809
	Males	1.80 ± 0.08 ^λ^	1.79 ± 0.07 ^λ^			
	Total	1.74 ± 0.09 ^λ^	1.75 ± 0.09 ^λ^			
Weight (kg)	Females	61.1 ± 5.5 ^λ^	58.1 ± 7.1 ^λ^	0.323	0.005	0. 014
	Males	70.2 ± 8.0 ^λ^	70.8 ± 9.4 ^λ^			
	Total	65.3 ± 8.1 ^λ^	66.4 ± 10.3 ^λ^			
Weekly training days	Females	5.9 ± 0.3	5.8 ± 0.5	0.594	0.936	0.839
	Males	5.9 ± 0.3	5.8 ± 0.8			
	Total	5.9 ± 0.3	5.8 ± 0.7			
Weekly strength training sessions	Females	3.5 ± 1.0 *	4.3 ± 1.7 *	0.084	0.244	0.213
	Males	3.5 ± 1.2	3.6 ± 1.4			
	Total	3.5 ± 1.1	3.9 ± 1.5			

Data expressed mean ± standard deviation (SD). ^λ^ Sex differences of the same competitive level; * Level differences of the same sex. Statistical significance fixed at *p* < 0.05.

**Table 2:** Number of SSs used by swimmers of both sexes and at different competitive levels according to AIS categories [21].

AIS Categories		Sex	Total	International	National	*p*-Value Level	*p*-Value Sex	*p*-Value Level-Sex
Total SS		Females	5.5 ± 3.5	6.4 ± 4.1	4.5 ± 2.4	0.091	0.841	0.600
		Males	5.4 ± 4.3	6.1 ± 4.8	5.1 ± 4.1			
		Total	5.4 ± 4.0	6.3 ± 4.4	4.9 ±3.6			
Group A	Sport foods	Females	1.4 ± 1.2	1.5 ± 1.4	1.3 ± 1.0	0.793	0.769	0.492
		Males	1.4 ± 1.2	1.3 ± 1.1	1.4 ± 1.2			
		Total	1.4 ± 1.2	1.4 ± 1.3	1.4 ± 1.1			
	Medical supplements	Females	1.0 ± 0.9	1.1 ± 0.89	1.0 ± 0.9 λ	0.012 *	0.210	0.049 *
		Males	0.7 ± 0.8	1.2 ± 1.0 ^#^	0.4 ± 0.6			
		Total	0.8 ± 0.9	1.2 ± 0.9 ^#^	0.6 ± 0.8			
	Sport performance	Females	1.0 ± 1.1	1.1 ± 1.2	0.8 ± 1.0 λ	0.979	0.160	0.190
		Males	1.4 ± 1.2	1.2 ± 1.0	1.5 ± 1.3			
		Total	1.2 ± 1.2	1.2 ± 1.1	1.3 ± 1.3			
	Total Group A	Females	3.4 ± 2.3	3.8 ± 2.6	3.1 ± 1.8	0.979	0.314	0.762
		Males	3.4 ± 2.5	3.7 ± 2.7	3.3 ± 2.4			
		Total	3.4 ± 2.4	3.7 ± 2.6	3.2 ± 2.2			
Group B		Females	0.9 ± 0.9	1.1 ± 0.9	0.6 ± 0.9	0.132	0.713	0.302
		Males	0.8 ± 0.9	0.8 ± 0.9	0.7 ± 0.9			
		Total	0.8 ± 0.9	1.0 ± 0.9	0.7 ± 0.9			
Group C		Females	1.2 ± 1.3	1.5 ± 1.4	0.8 ± 1.1	0.079	0.615	0.798
		Males	1.2 ± 1.9	1.6 ± 2.2	1.1 ± 1.8			
		Total	1.2 ± 1.7	1.6 ± 2.2	1.0 ± 1.6			

Data expressed mean ± standard deviation (SD). *: Significant difference for a determined factor; ^#^: Significant difference between international and national of a same sex; λ Significant difference between males and females of a same competitive level; Statistical significance at *p* < 0.05.

**Table 3:** Most used supplements according to sex and competitive level based on AIS categories [21].

AIS Categories		Supplement	Total	Sex				Level			
				Females	Males	*p*-Value	OR	International	National	*p*-Value	OR
Group A	Sport foods	Sport drinks	52.5%	46.2%	49.1%	0.542	0.72 [0.33–1.62]	46.2%	56.7%	0.410	0.66 [0.29–1.47]
		Sport bars	51.5%	45.1%	54.9%	0.685	0.80 [0.36–1.77]	43.6%	56.7%	0.223	0.59 [0.26–1.33]
		Whey protein	14.3%	31.6%	14.3%	0.059	2.77 [0.99–7.7]	33.3%	18.3%	0.099	2.2 [0.88–5.66]
	Medical supplement	Iron	33.3%	42.9%	26.3%	0.091	0.48 [0.20–1.11]	56.4%	18.3%	<0.001 *	5.77 [2.32–14.32]
		Vitamin D	27.3%	28.6%	26.3%	0.823	0.89 [0.37–2.18]	30.8%	25.0%	0.645	1.33 [0.54–3.27]
		Vitamin complex	22.2%	33.3%	14.0%	0.029 *	0.33 [0.12–0.87]	28.2%	18.3%	0.323	1.75 [0.67–4.55]
	Sport performance	Caffeine	53.5%	45.2%	59.6%	0.221	1.80 [0.80–4.01]	53.8%	53.3%	1.00	1.02 [0.46–2.29]
		Creatine	28.3%	16.7%	36.8%	0.041 *	2.92 [1.10–7.72]	25.6%	30.0%	0.820	0.81 [0.33–1.99]
		Bicarbonates	21.2%	14.3%	23.6%	0.214	2.14 [0.75–6.10]	15.4%	25.0%	0.319	0.55 [0.19–1.56]
		β-alanine	17.2%	21.4%	14.0%	0.421	0.60 [0.21–1.71]	20.5%	15.0%	0.587	1.46 [0.51–4.12]
Group B		Vitamin C	43.4%	40.4%	47.6%	0.540	0.74 [0.33–1.66]	53.8%	36.7%	0.102	2.02 [0.89–4.57]
		Vitamin E	16.2%	14.3%	17.5%	0.785	1.28 [0.42–3.84]	-	-	-	-
		Carnitine	13.1%	16.7%	10.5%	0.386	0.59 [0.18–1.90]	15.4%	11.7%	0.762	1.38 [0.43–4.45]
Group C		Magnesium	20.2%	21.4%	19.3%	0.805	0.88 [0.33–2.36]	28.2%	15.0%	0.129	2.23 [0.82–6.01]
		Glutamine	20.2%	23.8%	17.5%	0.459	0.68 [0.25–1.82]	25.6%	16.7%	0.313	1.72 [0.64–4.63]
		Royal jelly	20.2%	26.2%	15.8%	0.217	0.53 [0.20–1.42]	25.6%	16.7%	0.313	1.72 [0.64–4.63]
		Taurine	12.1%	-	21.0%	0.001 *	-	7.7%	15.0%	0.355	0.47 [0.12–1.87]

* Statistical difference in the consume between groups (*p* < 0.05).

## Data Availability

The data presented in this study are available on request from the corresponding author. The data are not publicly available due to privacy restrictions.
